# Decoupled form and function in disparate herbivorous dinosaur clades

**DOI:** 10.1038/srep26495

**Published:** 2016-05-20

**Authors:** Stephan Lautenschlager, Charlotte A. Brassey, David J. Button, Paul M. Barrett

**Affiliations:** 1 School of Earth Sciences, University of Bristol, Life Sciences Building, 24 Tyndall Avenue, Bristol BS8 1TQ, UK; 2 Faculty of Life Sciences, University of Manchester, Oxford Road, Manchester M13 9PL, UK; 3 School of Geography, Earth and Environmental Sciences, University of Birmingham, Edgbaston, Birmingham, B15 2TT, UK; 4 Department of Earth Sciences, The Natural History Museum, Cromwell Road, London SW7 5DB, UK

## Abstract

Convergent evolution, the acquisition of morphologically similar traits in unrelated taxa due to similar functional demands or environmental factors, is a common phenomenon in the animal kingdom. Consequently, the occurrence of similar form is used routinely to address fundamental questions in morphofunctional research and to infer function in fossils. However, such qualitative assessments can be misleading and it is essential to test form/function relationships quantitatively. The parallel occurrence of a suite of morphologically convergent craniodental characteristics in three herbivorous, phylogenetically disparate dinosaur clades (Sauropodomorpha, Ornithischia, Theropoda) provides an ideal test case. A combination of computational biomechanical models (Finite Element Analysis, Multibody Dynamics Analysis) demonstrate that despite a high degree of morphological similarity between representative taxa (*Plateosaurus engelhardti*, *Stegosaurus stenops*, *Erlikosaurus andrewsi*) from these clades, their biomechanical behaviours are notably different and difficult to predict on the basis of form alone. These functional differences likely reflect dietary specialisations, demonstrating the value of quantitative biomechanical approaches when evaluating form/function relationships in extinct taxa.

Morphologically, functionally and behaviourally similar traits are known to occur in unrelated taxa as a consequence of convergent evolution^1^. The independent evolutionary origins of these features are usually presumed to be triggered by similar functional demands or the occupation of comparable ecological niches. Classic examples of this phenomenon include the independent evolution of powered flight in birds, pterosaurs and mammals by modification of the forelimbs, and the development of a stream-lined, fusiform body shape in marine reptiles, marine mammals and cartilaginous and bony fish to optimise aquatic locomotion^2^,^3^.

Within Dinosauria, herbivory has been acquired multiple times independently in such disparate clades as Sauropodomorpha, Ornithischia and Theropoda^4^,^5^ and is one of the major factors that facilitated the diversification and later success of dinosaurs^6^,^7^. While dietary adaptation in dinosaurs culminated in the evolution of numerous unique morphological specialisations in derived groups, such as hadrosaurs or sauropods^8^,^9^, several other herbivorous clades exhibit convergence in craniodental morphology. This is most obvious in three phylogenetically distant groups: basal sauropodomorphs (‘prosauropods’), stegosaurs and therizinosaurs. The members of these groups are separated from each other both temporally and spatially, and are characterized by distinct, divergent bodyplans. While prosauropods include small to large-sized, bipedal, long-necked forms^10^, stegosaurs were large, heavily-built obligate quadrupeds equipped with extensive body armour^11^. In contrast, therizinosaurs were bipedal, long-necked, secondarily herbivorous theropods with elongate claws on their feathered forelimbs^12^,^13^. In spite of their different morphologies, members of these groups are united by their adoption of herbivorous (or partially omnivorous) diets, as evidenced by a suite of shared features in their cranial skeleton (Fig. 1). These include an elongate, mediolaterally narrow skull, a ventrally directed dentary symphysis and a ventrally displaced jaw joint. The small, lanceolate and coarsely denticulate teeth are medially inset, whereas the premaxilla is either edentulous and/or equipped with a keratinous rhamphotheca^11^,^14^,^15^.

It is tempting to use these parallel occurrences of morphological similarities in prosauropod, stegosaur and therizinosaur crania to infer functional similarity in feeding mechanics and behaviour^16^,^17^–a similarity that is particularly striking when contrasted with the divergent postcranial morphologies of these taxa^10^,^11^,^12^,^13^. However, although routinely invoked in the past, the inference of function from form is far from straightforward in extinct, and even extant, organisms. Convergent skeletal morphology is not in itself an indicator of functional convergence, as functional similarity can be achieved with differing morphologies^18^,^19^. Consequently, it is unclear whether stringent biomechanical constraints associated with the acquisition of herbivory in dinosaurs triggered the development and evolution of convergent morphologies, indicating limited plasticity, or whether cranial function is decoupled from superficially similar morphologies. The high degree of craniodental similarity in these otherwise divergent and phylogenetically disparate taxa provides a unique opportunity to assess the form/function relationship quantitatively. Because the qualitative assessment of convergent form and apparent function can be misleading, we employ a biomechanical approach to quantify functional similarities and differences. Here, we use Finite Element Analysis (FEA) in conjunction with Multibody Dynamics Analysis (MDA) to test the hypothesis that dietary adaptation resulted in convergent ecomorphological trajectories among disparate dinosaur clades. These two engineering techniques allow modelling of the deformation of geometric structures due to external loading and the simulation of dynamic behaviour of interconnected rigid bodies and are ideally suited for the study of musculoskeletal function^20^,^21^. Furthermore, we use these biomechanical modelling techniques to identify functional similarities and differences between these taxa that are not apparent from morphology alone, enabling us to investigate the palaeoecology of these extinct taxa. We apply these methods to three exemplar dinosaur taxa, each representing the independent acquisition of herbivory within a major clade: *Plateosaurus engelhardti*, a basal sauropodomorph from the Upper Triassic of Germany^22^, *Stegosaurus stenops*, a stegosaur from the Upper Jurassic of the USA^23^ and *Erlikosaurus andrewsi*, a therizinosaur from the Upper Cretaceous of Mongolia^15^,^24^.

## Results

### Bite forces

**Figure 2:**
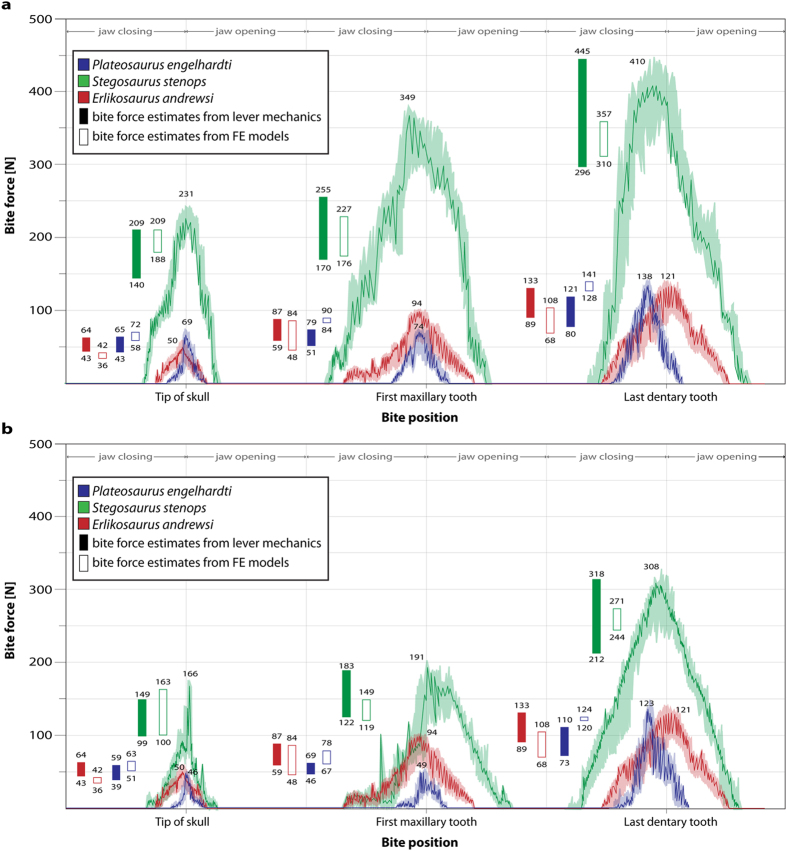
Bite force measurements for studied taxa recorded with multibody dynamics analysis. (**a**) All models in original size, (**b**) models scaled to same surface area. Bold lines represent recorded values during bite cycle with shaded background showing maximum and minimum values obtained during multiple analyses. Filled bars denote calculated bite force values derived from lever mechanic relations, open bars denote values obtained from reaction forces at bite points of the FE models.

Bite force measurements for the three dinosaur taxa were calculated using MDA for both the original sized cranial models (Fig. 2a) and for all models scaled to the same surface area to allow size-independent comparability (Fig. 2b). The highest bite forces are recorded in *Stegosaurus stenops*, both in the original (231–410 N) and scaled models (166–321 N): these values lie within the range of bite forces found by a previous study^25^. As expected, bite forces increase as the bite point shifts caudally (i.e. closer to the jaw joint). Bite forces in *Plateosaurus engelhardti* and *Erlikosaurus andrewsi* are found to be very similar to each other (*Plateosaurus*, 69–138 N; *Erlikosaurus*, 50–121 N) regardless of scaling (*Plateosaurus*, 46–123 N; *Erlikosaurus*, 50–121 N) and are consistently 60–75 percent lower than those for *Stegosaurus stenops* (unscaled: 231–410 N; scaled: 166–321 N). Independently obtained bite force estimates derived from the respective FEA models, as well as lever mechanic calculations, show a good correspondence with those obtained from the MDAs for all taxa and bite positions (Fig. 2). The only exception to this concerns the first maxillary tooth position in the originally-sized model of *Stegosaurus stenops*. In this example, bite forces obtained from FEA and lever mechanic models are only around 50–60 percent of the value obtained from the MDA model. Repeated analyses resulted in the same values suggesting that this is not a methodological artefact, but the reason for this outlier is unclear.

### Cranial stress distribution

**Figure 3:**
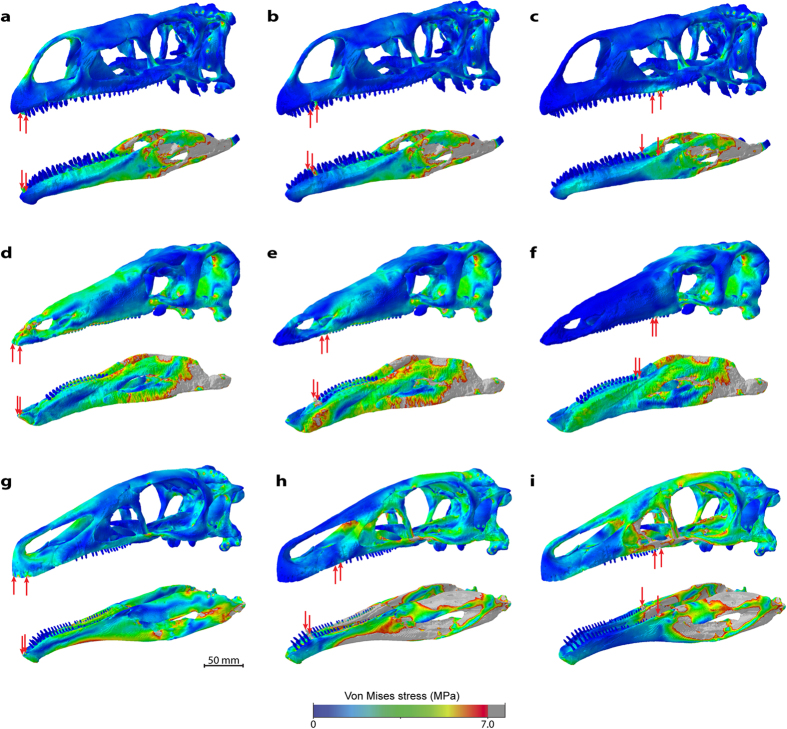
Comparison of Von Mises stress distribution for scaled models. Models of (**a–c**) *Plateosaurus engelhardti*, (**d–f**) *Stegosaurus stenops* and (**g–i**) *Erlikosaurus andrewsi* subjected to different bite scenarios. From left to right, bilateral bite at the tip of the skull/dentary, the first maxillary tooth/occluding tooth on dentary, last occluding maxillary/dentary tooth (indicated by red arrows). All models scaled to same surface area.

A comparison of the stress distributions over the skull and lower jaw models reveals considerable differences between the three taxa. The overall lowest stress magnitudes in the skull models are recorded for *Plateosaurus engelhardti*, which exhibits a largely uniform distribution of stresses for the different bite scenarios (Fig. 3a–c). Localised stress hotspots are restricted to the bite points, the premaxillary process in the two rostral bite scenarios and the muscle attachments (see Supplementary information for details on muscle origins and insertions). In contrast, the skull models of *Stegosaurus stenops* and, in particular, *Erlikosaurus andrewsi* are characterised by increased stress magnitudes. In *Stegosaurus stenops* stresses are centred on the rostral skull and the antorbital region (Fig. 3d–f), whereas in *Erlikosaurus andrewsi* large regions on the caudal part of the skull are relatively highly stressed (Fig. 3g–i), especially for the caudal bite scenario.

The lower jaw models across all taxa generally show higher stress magnitudes and less uniform stress distributions than the skull models (Fig. 3). As with the skull, the jaw models of *Plateosaurus engelhardti* display the lowest stress magnitudes. Stress is highest in the postdentary region and at the muscle insertions (Fig. 3a–c). Localised hotspots are found on the dentary lateral to the tooth row, although not along the lateral dentary shelf, during the rostral biting scenarios, and at the tip of the dentary for a bite at the caudal-most tooth position. In comparison, *Stegosaurus stenops* shows high stress magnitudes across the entire jaw, with a focus on the postdentary and articular regions (Fig. 3d–f). The highest stress magnitudes are recorded in *Erlikosaurus andrewsi*, in particular for a bite in the rostral region of the tooth row (Fig. 3h). In contrast, the bite at the caudal-most tooth position shows a distinct pattern of stress distribution with high magnitudes in the postdentary bones and a largely unstressed dentary region (Fig. 3i).

In addition to the osteological models, further models were analysed that incorporated a keratinous beak covering the rostral regions of the skull and dentary in *Stegosaurus stenops* and *Erlikosaurus andrewsi* (Supplementary Fig. S4). As demonstrated in previous analyses, the presence of a keratinous sheath has stress mitigating effects on the underlying bone^26^ and this observation was confirmed here. This effect is more pronounced in the skull models and for the two rostral biting regimes. The overall stress distribution remains largely unchanged, although individual stress magnitudes decrease.

*Stegosaurus stenops* differs notably from the other taxa examined in the absence of an antorbital fenestra^22^. To rule out the possibility that its absence obscures functional similarities, a hypothetical skull model was created to incorporate this feature (see Materials and Methods). However, analyses of the different bite scenarios display only negligible differences in terms of the stress distributions between the actual and hypothetical models of *Stegosaurus stenops* (Supplementary Fig. S5).

**Figure 4:**
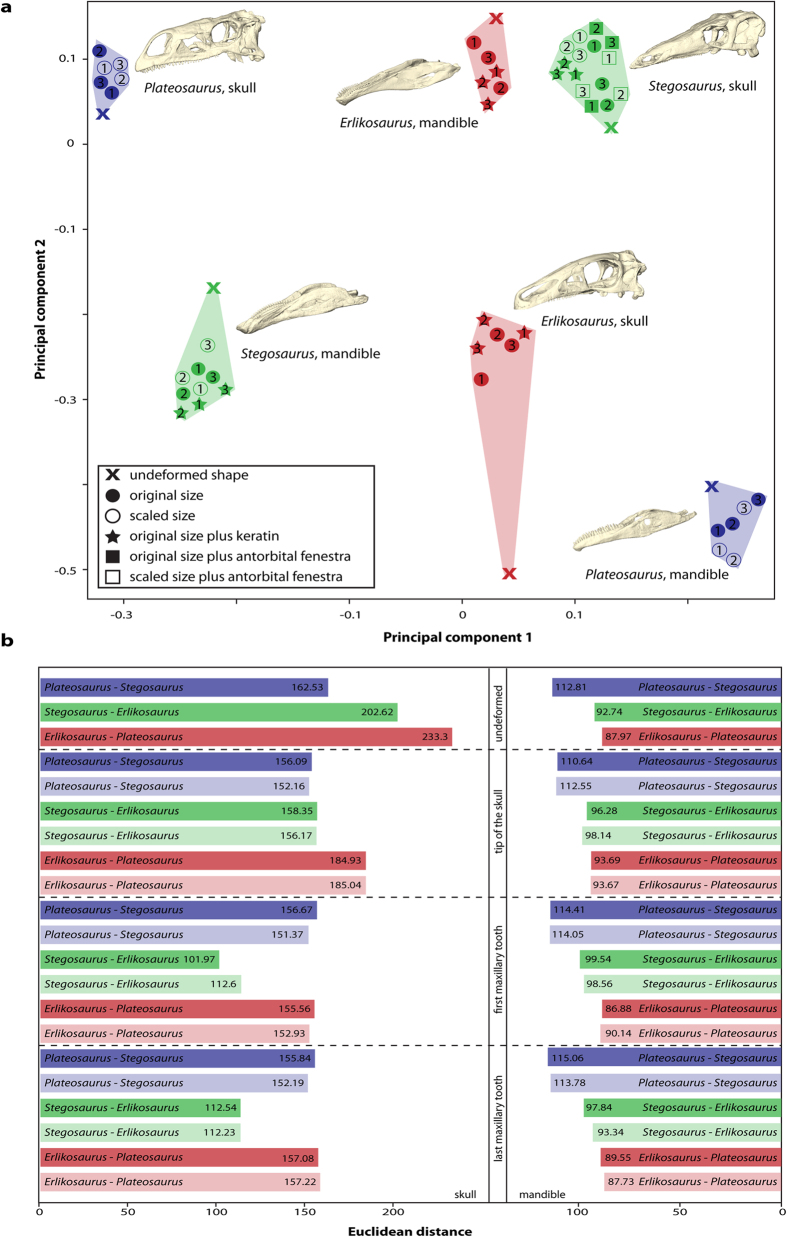
Quantitative assessment of biomechanical differences. (**a**) Principal component plot showing extent of deformation of models during biting simulations using FEA. Skull and lower jaw models plotted into the same coordinate system. Numbers indicate bite position (corresponding to Fig. 3): 1, bite at the tip of the skull/dentary, 2, the first maxillary tooth/occluding tooth on dentary, 3, last occluding maxillary/dentary tooth. (**b**) Calculated Euclidean distances between studied taxa for undeformed models and for different bite scenarios. Pale background colours indicate models scaled to same surface area.

A geometric morphometric analysis of the results from all FEAs quantitatively confirms these observations (Fig. 4a). Deformation as a result of loading is most pronounced in the skull of *Erlikosaurus andrewsi*, whereas *Stegosaurus stenops* and *Plateosaurus engelhardti* show little variation from the undeformed/unloaded shape. For the lower jaw models, the differences between the taxa are more uniform with the highest variation found in *Stegosaurus stenops*. Calculation of Euclidean distances further demonstrates that the functional behaviour of the skull and the mandible are not consistent across the tested bite scenarios (Fig. 4b). While the skulls of *Plateosaurus engelhardti* and *Stegosaurus stenops* are most similar in their deformation pattern for a bite point at the tip of the skull, *Stegosaurus stenops* and *Erlikosaurus andrewsi* are more similar to each other in the other bite scenarios. In contrast, the deformation patterns of the mandibles indicate a closer functional similarity between *Erlikosaurus andrewsi* and *Stegosaurus stenops*, whereas *Plateosaurus engelhardti* and *Stegosaurus stenops* are the most dissimilar.

### Plant stress distribution

**Figure 5:**
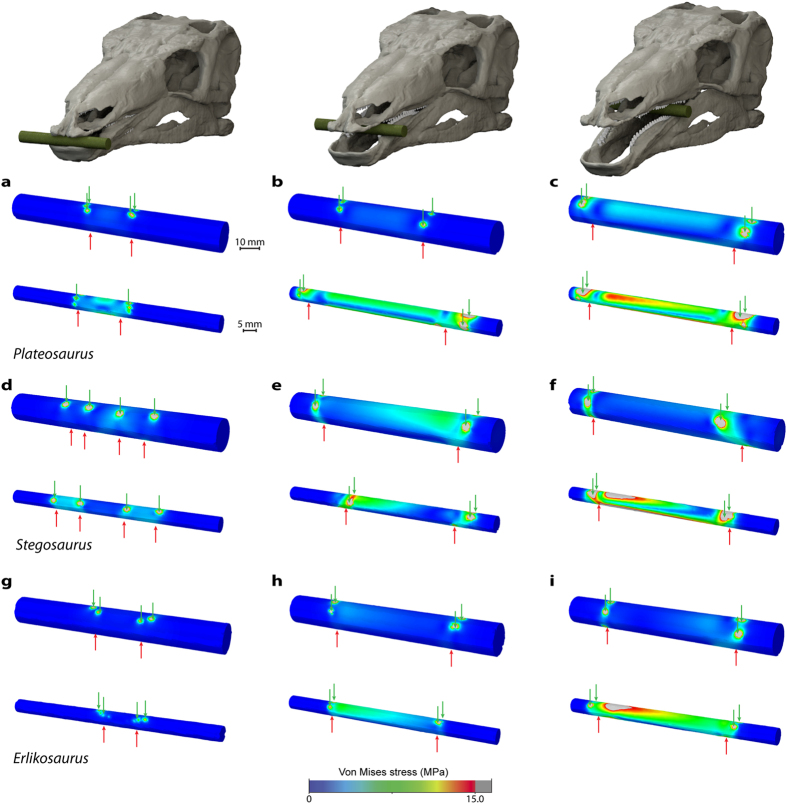
Comparison of Von Mises stress distribution for different sized plant models. Models of large (10 mm diameter) and small (5 mm diameter) plant items for (**a–c**) *Plateosaurus engelhardti*, (**d–f**) *Stegosaurus stenops* and (**g–i**) *Erlikosaurus andrewsi* subjected to different bite scenarios. From left to right, bite at the tip of the skull/dentary, the first maxillary tooth/occluding tooth on dentary, last occluding maxillary/dentary tooth (loads indicated by red arrows, constraints indicated by green arrows). All models scaled to same surface area.

The effects of bite force and position on different sized food items were tested for all taxa. For the larger plant model (10 mm in diameter), the stress distribution for all bite positions is largely similar for the scaled skull models and shows only low stress magnitudes (Fig. 5). However, for *Stegosaurus stenops* and *Plateosaurus engelhardti* the stress magnitudes are highest in the caudal bite scenario. The smaller plant models (5 mm in diameter) display more variable stress distributions. While a bite at the tip of the skull produces very low magnitudes, stress increases with a shift of the bite point (and coincident increase in bite force) caudally. Among these three taxa, stress magnitudes are highest for *Plateosaurus engelhardti* for each bite scenario across the width of the plant model. For all taxa, contour plots of the smaller plant models show marked asymmetries in stress distribution. This can be explained by the fact that the tooth positions, orientations and eruption stages, and thus the force vectors of the left and right sides of the skull and jaw, are subject to natural variation.

## Discussion

Results of the combined FEA and MDA simulations demonstrate that there are no consistent patterns in bite forces or stress distributions for the skull and lower jaw models of the studied taxa. While bite forces are very similar in *Erlikosaurus andrewsi* and *Plateosaurus engelhardti* they are considerably higher in *Stegosaurus stenops* (regardless of scaling), indicating that the skull morphology of the latter allowed for both a larger muscle mass and a more efficient conversion of muscle force into bite force. Considered on its own, this similarity in bite forces between *Plateosaurus engelhardti* and *Erlikosaurus andrewsi* could suggest an underlying phylogenetic signal, as the distribution of bite forces generally reflects phylogenetic relationships among the three studied taxa. The cranial stress distributions, however, show a different pattern with the skulls of *Stegosaurus stenops* and *Plateosaurus engelhardti* more similar to each other than to *Erlikosaurus andrewsi* in terms of their biomechanical responses to muscle loading. Quantified deformation patterns of the three taxa based on the calculation of Euclidean distances confirms that functional similarities are variable according to bite position (Fig. 4). This is unexpected, as *Stegosaurus stenops* and *Erlikosaurus andrewsi* share a number of similar anatomical features, such as general skull shape, the edentulous premaxillary region, the presence of a keratinous rhamphotheca and a U-shaped symphyseal morphology. In contrast, the loading regimes simulated for all three taxa result in less divergent stress distributions in the plant biting models. This suggests that despite the differences in bite forces and cranial stress resistance, all taxa have a similar efficiency in terms of processing potential food items

Although the bite forces reported herein are low in comparison to those of carnivorous dinosaurs^27^,^28^ they fall within the known range of extant herbivores (mean value around 88–141 N^29^) demonstrating that these bite forces were likely sufficient for dealing with vegetation in the studied taxa. However, the differences in absolute bite force values between these taxa are likely indicative of different feeding/foraging strategies and ecological niche occupations^30^*. Plateosaurus engelhardti* has often been considered an unspecialised herbivore or omnivore^10^ and the high cranial robustness displayed in the different bite scenarios could be interpreted as reflective of this generalist behaviour. Moreover, its low bite forces would have limited dietary selection to soft vegetation with limited oral processing^31^, or the gathering of more fibrous vegetation.

By contrast, *Erlikosaurus andrewsi* is characterised by high susceptibility to stress and deformation, particularly for loading regimes simulating biting along the tooth row. This indicates a feeding behaviour that was specialised to exploit the beak-like tip of the skull. As previously suggested, *Erlikosaurus andrewsi* may have recruited the postcranial musculature to compensate for these low bite forces and to relieve stresses on cranial structure consistent with an assumed herbivorous diet^5^,^26^,^32^. *Stegosaurus stenops*, on the other hand, possessed a combination of relatively high bite forces and only moderate stress magnitudes under any bite scenario, which indicates that it would have been capable of foraging on a wide variety of different vegetation types and plant matter, although other aspects of craniodental anatomy indicate that significant oral processing was unlikely^33^. These results suggest that in spite of morphological similarities, *Stegosaurus stenops* had access to a greater range of potential food plants than the other studied taxa. As demonstrated in previous studies that used tooth wear^34^, beak shape^35^ and muscle arrangement^36^ as an indicator, quantitative consideration of functional properties and biomechanical analyses can reveal many subtleties in trophic interactions and niche partitioning among herbivorous dinosaurs that are often overlooked by qualitative comparisons.

These previously unrecognised functional differences and the novel feeding specialisations inferred for all three taxa would have been difficult to identify on the basis of comparative morphology alone. A qualitative assessment of their crania has previously led to assumptions of functional convergence, in particular between the superficially similar skulls of *Stegosaurus stenops* and *Erlikosaurus andrewsi*, which share several functionally important characteristics, such as overall skull shape, an edentulous premaxilla, a rostral rhamphotheca and similar tooth morphology. Indeed, *Erlikosaurus andrewsi* (and therizinosaurs in general) had previously been compared morphologically and functionally to prosauropods and ornithischians due to craniodental similarity^16^,^17^. Data from this study, however, show that despite a striking number of common, but independently acquired craniodental adaptations, function is difficult to predict on the basis of shared form alone.

The biomechanical analyses revealed distinct differences in bite force magnitudes and stress resistance, suggesting a disparity between morphological and functional convergence within the resolution of the studied taxa. Possible future studies including a phylogenetically broader and morphologically more diverse set of dinosaurian herbivores (i.e. ceratopsians and ornithopods), however, could reveal patterns of gross morphofunctional convergence between basal sauropodomorphs, stegosaurs and therizinosaurs. When contrasted with other craniodental morphologies, these clades would most likely be more similar to each other than to morphologically more disparate herbivorous dinosaurs. While apparent morphological differences are more likely to result in functional differences, the results of this study show that extreme care should be taken when invoking simplistic form/function relationships in fossil taxa.

## Methods

### Specimens and digital reconstructions

Digital skull and lower jaw models of the three dinosaur taxa were created on the basis of CT scans of the original specimens: *Plateosaurus engelhardti* (MB.R.1937, Museum für Naturkunde, Berlin, Germany; skull length: 350 mm), *Stegosaurus stenops* (NHMUK PV R36730, Natural History Museum, London, UK; skull length: 375 mm) and *Erlikosaurus andrewsi* (IGM 100/111; Geological Institute of the Mongolian Academy of Sciences, Ulaan Bataar, Mongolia; skull length: 265 mm). Datasets were imported into Avizo (versions 6.3.1 and 7, FEI Visualization Science Group) for image segmentation and digital restoration to correct for taphonomic artefacts (see Supplementary information for scanning parameters and details of the reconstruction process). For *Erlikosaurus andrewsi* and *Stegosaurus stenops* the presence of a keratinous beak has been inferred based on the morphology of the edentulous premaxilla and tip of the dentary. To test the mechanical effects of a beak, additional skull models were created with a keratinous sheath covering the tip of the skull and dentary^26^. A further hypothetical skull configuration of *Stegosaurus stenops* was created incorporating an antorbital fenestra. This feature has been lost or reduced in many stegosaurs and other ornithischian dinosaurs. However, it has been shown that the presence of an antorbital feature can considerably affect biomechanical performance in archosaurs^37^. Consequently, a hypothetical model with antorbital fenestrae was created to test for possible stress-dissipating effects. The size and position of the antorbital fenestra were modelled after the condition present in the basal stegosaur *Huayangosaurus taibaii*^38^.

Three-dimensional reconstructions of the jaw adductor musculature were created for all taxa following the approach outlined by Lautenschlager^39^. Digital models of each muscle group were reconstructed on the basis of osteological correlates for muscle origin and insertion sites (Supplementary Fig. S1). Muscle dimensions and volumes were modelled according to spatial constraints within the adductor chamber and topological criteria (see Supplementary information for full details of the muscle reconstruction). Physiological cross-sectional area for each muscle was estimated by dividing the volume of each muscle by its total length to calculate muscle forces following Thomason^40^. (Supplementary Table S1).

### Biomechanical analysis

For FEA, all models were imported into Hypermesh 11 (Altair Engineering) for the creation of solid mesh FE models and the setting of boundary conditions. The skull models consist of approximately 2,000,000 tetrahedral elements and the lower jaw models of approximately 1,000,000 elements (for details see Supplementary Table S2). Material properties for cranial bone, enamel and dentine were assigned in Hypermesh based on available extant analogues: alligator mandible (E = 20.49 GPa, ʋ = 0.40), crocodile teeth (E = 60.40 GPa, ʋ = 0.31) and avian beak keratin (E = 1.04 GPa, ʋ = 0.40)^25^. All materials were treated as isotropic and homogenous, as anisotropy cannot be reliably measured in fossilised tissues. Although this assumption might affect the absolute magnitudes of the results, validation studies have demonstrated that this approach reliably predicts stress and strain patterns in comparative scenarios, as long as boundary conditions are consistent between models^41^. The skull models were constrained from translation in the x-, y- and z-axes at the paroccipital processes, the occipital condyle and the quadrates (15 nodes spread across these regions); jaw models were constrained at the glenoid (five nodes corresponding to the contact points with the quadrate). To simulate biting at different analogous positions, additional constraints (one node each) were applied to the tip of the premaxilla (*Stegosaurus* and *Erlikosaurus*) and the first premaxillary tooth (*Plateosaurus*), the first maxillary tooth and the caudal-most maxillary tooth with a counterpart present in the dentary. In the lower jaw models, constraints were applied at corresponding positions to the tip of the predentary (*Stegosaurus*), the dentary (*Erlikosaurus*) or the first premaxillary tooth (*Plateosaurus*), the first dentary tooth occluding with the first maxillary tooth position and the last dentary tooth. In addition to the original sized models, a second set of simulations was run with all models scaled to the same surface area (see also Supplementary Tables S3–S5). Muscle forces were scaled proportionally to surface area. All models were subsequently imported into Abaqus 6.10 (Simulia) for analysis and post-processing.

For MDA the digitally restored skull and lower jaw models were imported into ADAMS 2013 (MSC Software Corp.) as rigid bodies in parasolid format. The skull was kept fixed throughout the simulations, whereas the lower jaw was modelled as a mobile element connected to the skull by a hinge joint at the quadrate/articular contact. Mass and inertial properties were calculated in ADAMS based on rigid body geometry and a standard tissue density of 1050 kg/m^3 ^42. Adductor muscles were modelled as springs connecting corresponding attachment sites. Additional depressor muscles were incorporated into the model to allow dynamic jaw opening and closing (Supplementary Fig. S2). Muscle forces were assigned according to the calculations taken from the three-dimensional reconstructions. All muscles were then activated applying the dynamic geometric optimization (DGO) method^43^. To simulate biting, hypothetical and simplified food items (‘plant’ models) were modelled in ADAMS with a thickness of 5 and 10 mm–dimensions consistent with the vegetation foraged by herbivorous mammals^44^–and placed perpendicular to the teeth at the aforementioned tooth positions by moving them in a rostrocaudal direction during jaw opening phases. Bite force measurements were recorded throughout the bite cycle (Fig. 2). Bite force estimates were independently calculated for all three taxa using two-dimensional lever mechanic relationships^39^ as well as reaction forces obtained from the cranial FE models.

To assess cranial efficiency during biting, complementary FE models of the different sized, hypothetical plant models (5 and 10 mm in diameter) used for the MDA were created following a similar approach to that of Reichel^25^. This approach was taken to evaluate the bite force transfer and stress distribution in the three taxa. Material properties (E = 11.04 GPa, ʋ = 0.33; default properties for wood in ADAMS) and boundary conditions, such as tooth contact positions and bite force, were exported from the MDA simulations into Hypermesh. The plant models were constrained at the contact with the skull, loaded at the contact points with the lower jaw with the respective bite forces and were subsequently imported into Abaqus for solving.

Biomechanical performance for the cranial and plant FE models were assessed via contour plot outputs (Figs 3 and 5). In addition, the resulting deformation models of the skull and lower jaw were subjected to a geometric morphometric analysis. Consequently, 35 landmarks from each of the skull models and 24 landmarks from the lower jaw models (including undeformed shape) were recorded (see Supplementary Table S6, Supplementary Fig. S3). Landmark sets were subjected to Procrustes superimposition and a principal component analysis (PCA) performed in PAST^45^; Euclidean distances for the undeformed and deformed skull and jaw shapes were calculated in PAST to provide a quantitative measure of morphological and functional differences.

## Additional Information

**How to cite this article**: Lautenschlager, S. *et al.* Decoupled form and function in disparate herbivorous dinosaur clades. *Sci. Rep.* **6**, 26495; doi: 10.1038/srep26495 (2016).

## Supplementary Material

Supplementary Information

## Figures and Tables

**Figure 1:**
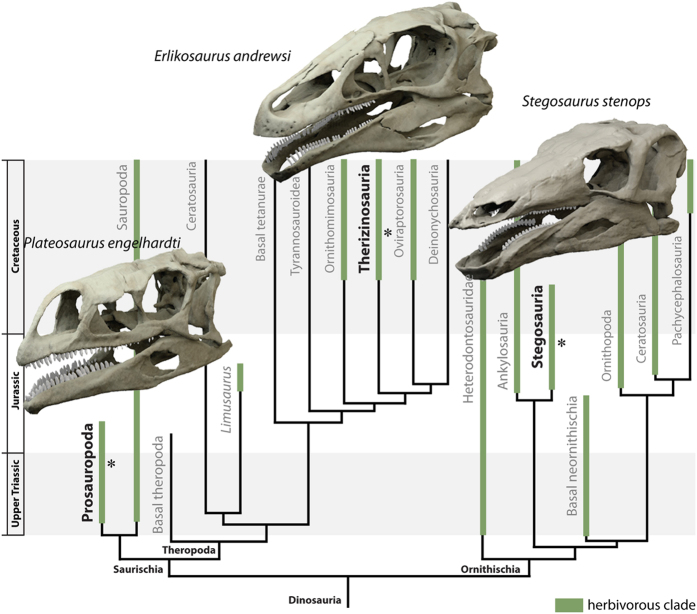
Digitally restored models of *Plateosaurus engelhardti*, *Stegosaurus stenops* and *Erlikosaurus andrewsi* in their phylogenetic and stratigraphic context. Asterisk denotes stratigraphic position and clades with herbivorous members are highlighted in green. Phylogeny simplified from^7^.
