# Supplementary material for: Decoupled form and function in disparate herbivorous dinosaur clades
Source: Sci Rep. 2016 May 20;6:26495. doi: 10.1038/srep26495 (PMC4873811; doi:10.1038/srep26495)
Supplement: Supplementary Information [file srep26495-s1.pdf]

Supplementary information

Decoupled form and function in disparate herbivorous dinosaur clades

Stephan Lautenschlager[1,*], Charlotte A. Brassey[2], David J. Button[3], Paul M. Barrett[4]

[1]School of Earth Sciences, University of Bristol, Life Sciences Building, 24 Tyndall Avenue, Bristol BS8 1TQ, UK

[2]Faculty of Life Sciences, University of Manchester, Oxford Road, Manchester M13 9PL, UK

[3]School of Geography, Earth and Environmental Sciences, University of Birmingham, Edgbaston, Birmingham, B15 2TT

[4]Department of Earth Sciences, The Natural History Museum, Cromwell Road, London SW7 5DB, UK

[*]Corresponding author: glzsl@bristol.ac.uk

TABLE OF CONTENTS

SCANNING PARAMETERS FOR STUDIED SPECIMENS 3

Plateosaurus engelhardti 3

Stegosaurus stenops 3

Erlikosaurus andrewsi 4

DIGITAL RESTORATION OF SPECIMENS 5

Plateosaurus engelhardti 5

Stegosaurus stenops 6

Erlikosaurus andrewsi 6

MUSCLE RECONSTRUTION 7

Plateosaurus engelhardti 7

Stegosaurus stenops 12

ADDITIONAL DATA AND FIGURES FOR BIOMECHANICAL ANALYSES 18

SUPPLEMENTAL REFERENCES 33

SCANNING PARAMETERS FOR STUDIED SPECIMENS

Plateosaurus engelhardti

CT scans of the skull and both mandibles of *Plateosaurus engelhardti* (MB.R.1937; Humboldt Museum für Naturkunde, Berlin) were generously provided by Dr R. Goessling, on behalf of the Humboldt Museum für Naturkunde. The specimens were originally scanned at the Faculty of Veterinary Medicine, Freie Universität, Berlin using a GE Medical Systems LightSpeed QX/I CT scanner. Scan parameters were set at 120 kV and 330 mA, yielding a stack of 281 slices for the skull and 273 for each mandible with a slice thickness of 1.25 mm.

The skull and both mandibles of *Plateosaurus engelhardti* (MB.R.1937; Humboldt Museum für Naturkunde, Berlin) were CT scanned by R. Goessling and the Humboldt Museum für Naturkunde as part of an unconnected study. Specimens were scanned at the Faculty of Veterinary Medicine, Freie Universität, Berlin using a GE Medical Systems LightSpeed QX/I Ct scanner. Scan parameters were set at 120 kV and 330 mA, yielding a stack of 281 slices for the skull and 273 for each mandible with a slice thickness of 1.25 mm. This scan data was then provided to the current working group by L.M. Witmer with the generous permission of R. Goessling on behalf of the Humboldt Museum für Naturkunde.

Stegosaurus stenops

The skull of *Stegosaurus stenops* (NHMUK PV R36730) was CT scanned at the Natural History Museum, London, U.K., using a Metris (now Nikon Metrology) HMX ST 225 CT scanner. The skull consisted of disarticulated isolated elements which were scanned separately. Scan parameters therefore ranged from 180-220 kV at 160 mA, using copper filters of 0.25-2.5 mm in thickness. Resulting voxel sizes ranged from 20-97 μm. Scans were reconstructed in CT Pro (Nikon

Metrology, U.K.) and exported from VG Studio Max (Volume Graphics, Heidelberg, Germany) as .vol files.

Erlikosaurus andrewsi

The skull of *Erlikosaurus andrewsi* (IGM 100/111; Geological Institute of the Mongolian Academy of Sciences, Ulaan Bataar, Mongolia) was CT scanned at XTek Systems Ltd. (now Nikon Metrology), Tring, Hertfordshire, U.K., using a XT-H-225ST CT scanner. Scan parameters were set at 180 kV and 145 mA for the complete skull. Additional scans were performed for the braincase region at 180 kV and 135 mA. The resulting rotational projections were processed with custom-built software provided by X-Tek Systems Ltd. Creating a VGI and a VOL file, containing 1998 slices with a slice thickness of 145 μm for the complete skull and 1000 slices with a slice thickness of 108 μm for the braincase region.

DIGITAL RESTORATION OF SPECIMENS

Plateosaurus engelhardti

Scan data of MB.R.1937 were imported into Avizo (versions 6.3.1 and 7, FEI Visualization Science Group). Individual cranial elements were segmented utilising the Avizo segmentation editor, with manual removal of cracks and small breaks. MB.R.1937 has suffered lateromedial compression and shearing, with the right side in particular being displaced dorsally and medially. Cranial reconstruction was hence based primarily on elements from the left side of the skull, apart from the descending process of the postorbital, ascending process of the jugal and paraoccipital process of which the right-side element was considered better preserved. Each element, post repair, was mirrored across the bilaterally symmetrical long axis of the skull to produce their antimere. The proportions of each element and of the completed skull and mandible models were compared throughout to those of other *Plateosaurus* specimens and pre-existing reconstructions[S1-S6] to ensure consistency.

Reconstruction and rearticulation was performed in systematic order with the least deformed bones- the left frontal, parietal, squamosal, quadrate and maxilla- restored first. Restoration of the skull roof allowed rearticulation of the displaced braincase. The maxillae allowed rearticulation of the premaxillae after repair of the damaged ascending process and repair of warpage to provide a flat midline surface for articulation with the opposing premaxilla. These completed skull roof and snout regions then provided greater constraint on the remaining facial and palatal bones. The pterygoids of MB.R.1937 have been lateromedially crushed and buckled; these were restored last so that surrounding bones of the skull could be used to aid in reconstruction of their original proportions. The epipterygoids of MB.R.1937 are heavily fragmented; these were hence reconstructed after those of AMNH FARB 6810 (American Museum of Natural History, New York) [S6]. Additionally, the orbitosphenoids of MB.R.1937 are entirely absent and so were manually

reconstructed after those of other sauropodomorphs. The mandibles of MB.R.1937 required less reconstruction although the dentaries have suffered some lateromedially flattening and cracking, these were repaired using the curvature of the upper toothrow as a guide.

Stegosaurus stenops

For the restoration process of NHMUK PV R36730, surface models of the individual elements obtained from CT scanning were imported as .ply files into Avizo. Small cracks and fractures were removed manually by using the paintbrush tool in Avizo's segmentation editor. Retrodeformation of selected elements, such as the articulated braincase, was performed using the geometric morphometrics software Landmark (version 1.6, www.idav.ucdavis.edu/research/EvoMorph[S7]). Missing elements (left jugal, left supraorbital2, right supraorbital1, right angular, right articular) on one side of the skull were reflected along the bilateral symmetry plane. Elements, which had not been preserved (palatines, vomer, predentary) were modelled manually after published examples[S8,S9] and comparisons with other specimens (USNM 4934, United States National Museum' Washington, D.C.). The articulation of the final model was performed on the basis of evidence provided by articular facets, the size and spatial extent of the individual elements.

Erlikosaurus andrewsi

For the restoration of the skull of *Erlikosaurus andrewsi* the individual skull elements were segmented as separate materials in Avizo. Small crack, breaks and holes were removed by interpolating over the affected region. As with the digital models of *Plateosaurus engelhardti* and *Stegosaurus stenops,* the bilateral symmetry was exploited to restore incomplete (both lacrimals, right frontal) or partially missing elements (left nasal). Finally, the individual elements were articulated, following the information provided by undeformed regions of the skull or as indicated by sutures and articulation facets on each element[S10].

MUSCLE RECONSTRUTION

The jaw adductor musculature for the three studied taxa were reconstructed following the protocol laid out by Lautenschlager[S11]. Digital models of each muscle group were reconstructed on the basis of osteological correlates for muscle origin and insertion sites[S12]. Muscle dimensions and volumes were modelled according to spatial constraints within the adductor chamber and topological criteria. As a full account of the adductor muscle reconstruction for *Erlikosaurus andrewsi* has previously been published the reader is referred to the respective publication[S11].

Plateosaurus engelhardti

m. adductor externus superficialis (m. AMES)

The attachment site of the m. AMES on the temporal bar is consistent across sauropsids, although it rarely leaves a specific osteological correlate beyond a generally smooth surface on the postorbital and squamosal borders of the supratemporal fenestra[S12,S13]. In *Plateosaurus engelhardti* the medial surfaces of the postorbital and squamosal along the edge of the bar are generally smooth, allowing the reconstruction of the m. AMES attachment here as a level I inference. Rostrally, this attachment area is constrained by the position of the m. PSTs. Caudally, the attachment site extends into the caudal corner of the supratemporal fenestra, on the main body of the squamosal. The mediolateral width of the m. AMES is constrained by the other muscles of the adductor externus group, rather than osteological features.

The insertion of the m. AMES on the surangular is likewise highly conserved across sauropsids, where it occupies the dorsolateral edge of the surangular[S12,S13]. In *Plateosaurus engelhardti* this insertion site is marked by a smooth, dorsomedially bevelled region. This also makes the insertion site of the m. AMES a level I inference.

m. adductor externus medialis (m. AMEM)

The m. AMEM is somewhat problematic as it is generally difficult (or impossible) to discern from the m. AMEP and m. AMES in sauropsids[S12,S13]. As a result, identification of its attachment sites depends heavily on the topology of other reconstructed muscles, especially for its insertion site on the mandible[S11,S12].

The m. AMEM originates along the caudal wall of the supratemporal fenestra in archosaurs[S12,S13], attaching along the rostral face of the parietal wing and medial process of the squamosal. This attachment is marked by a large, smooth region. The rostromedial boundary of the m. AMEM is defined by the relative position of the m. AMEP; a slight scar marks the distinction between the two muscle groups[S12]. These correlates make the insertion area of the m. AMEM a level I inference for both taxa.

The m. AMEM inserts onto the dorsomedial edge of the surangular in sauropodomorphs[S12]. This area is narrow, smooth and slightly concave. This area is continuous with the insertion site of the m. AMEP, which extends from the dorsomedial edge of the surangular onto the coronoid area in both taxa. Distinguishing between the two is difficult; two smooth, slightly concave areas are observed with a weak break between them. This break is taken here as the distinction between the insertion sites of these two muscles, with the m. AMEM attachment site running from here until the dorsomedial edge of the surangular pinches out caudally. Nevertheless, the ambiguous nature of this distinction, and the lack of a specific correlate observed for this attachment in extant crocodilians and birds[S12], renders this reconstruction a level I' inference.

m. adductor mandibulae externus profundus (m. AMEP)

This muscle occupies the rostromedial area of the supratemporal fenestra in sauropsids[S12,S13]. It originates on the parietal rostral to the attachment of the m. AMEM. In *Plateosaurus engelhardti*, it chiefly occupies the lateral surface of the main body of the parietal. It is bounded laterally by the m.

PSTs, which occupies the rostroolateral wing of the parietal. The prominent smooth regions on the parietal marking the attachment of this muscle make this origination site a level I inference for both taxa.

In extant sauropsids the m. AMEP attaches in the region of the coronoid eminence, rostral to the attachment of the m. AMEM (in those cases where it can be distinguished from the latter muscle)[S12,S13], making such an attachment a level I inference in sauropodomorphs. Hence, here it is reconstructed as attaching to the dorsomedial surface of the rostralmost surangular and caudal coronoid in *Plateosaurus engelhardti*. Further rostral expansion of the m. AMEP is prevented by the ectopterygoid, which tightly constrains both the size and attitude of this muscle.

m. pseudotemporalis superficialis (m. PSTs)

The m. PSTs is the deepest and most rostral of the temporal muscles, originating from the rostral wall of the supratemporal fenestra in archosaurs[S12,S13]. Reconstructed it as originating here is a class I inference, although the generally smooth surface of the supratemporal fossa makes its attachment site hard to distinguish from those of the m. AMEP and m. AMES. It is here reconstructed as occupying the majority of the rostrolateral parietal wing, the caudal wall of the laterosphenoid and the frontal. The frontal portion of the supratemporal fossa in *Plateosaurus engelhardti* is deep; this is not a taphonomic artefact as it is preserved on both sides and is also seen in other *Plateosaurus* specimens (e.g. AMNH FARB 6810; S6]). Similar deep fossae are present on the frontals of some other ‘prosauropods’ and theropods, where it has also been reconstructed as representing the extent of m. PSTs attachment[S14,S15], although Holliday[S12] considered such an attachment in these theropods unlikely, partially due to the strong horizontal orientation of the fossa. However, due to its close association with the supratemporal fossa and caudodorsal orientation in *Plateosaurus engelhardti*, this fossa is reconstructed here as being occupied by the m. PSTs, as in previous reconstructions of *Plateosaurus*[S2,S16].

The mandibular insertion of the m. PSTs is somewhat problematic in dinosaurs[S12], Galton S2 and Fairman S16] reconstructed an insertion onto the medial region of the coronoid in *Plateosaurus*, through comparison with lepidosaurs. However, phylogenetic bracketing[S12], the large-size of the adductor fossa and the small size of the coronoid eminence instead suggest insertion within the rostral mandibular adductor fossa, similar to the condition seen in crocodiles and most ratites[S12,S13]. Still, the variability of this attachment site in birds, and the lack of a specific osteological correlate for m. PSTs attachment, render this a level II' inference. An attachment in the region of the coronoid eminence would also lead to problems regarding spatial relationships with the other adductor muscles as the adductor chamber is very narrow.

In *Plateosaurus engelhardti* the mandibular adductor fossa is strongly laterally compressed. This spatial constraint suggests a tendinous, rather than fleshy, attachment of this muscle[S11], as in extant crocodilians[S12,S13,S17,S18]. Additionally, the enlarged and well-developed pterygoid flange of *Plateosaurus engelhardti* tightly compresses the pathway for the m. PSTs. This is similar to the morphology seen in extant crocodilians where the compressive environment is associated with the development of a sesamoid (the 'cartilago transilisens') within the m. PSTs[S17]. The development of similar fibrocartilaginous structure within the m. PSTs in *Plateosaurus engelhardti* is therefore tenable.

m. pseudotemporalis profundus (m. PSTp)

Phylogenetic bracketing suggests that m. PSTp would have originated from the lateral wall of the epipterygoid in those dinosaurs that possessed the bone[S12] even though distinct osteological correlates are rare. The more basally branching *Plateosaurus engelhardti* retained an epipterygoid; the m. PSTp is here reconstructed as originating on the expanded rostrolateral surface of the epipterygoid, dorsal to the midshaft.

The mandibular attachment of the m. PSTp is also difficult to discern due to the ambiguous nature of osteological correlates for this muscle and its typically vestigial development in extant archosaurs[S12]. Topological relationships with other muscles, in particular the m. PSTs, and the small area of attachment on the mediodorsal edge of the surangular, make an rostroventral attachment within the mandibular adductor fossa adjacent to that of the m. PSTs seem more likely than attachment on the medial surface of the coronoid region[S16], as in squamates and most birdsS12,S13. Although the m. PSTs is herein considered to be similar to that of extant crocodilians, in these taxa the m. PSTp merges into the m. PTd rather than inserting onto the mandible itself[S13,S18].

m. adductor mandibulae posterior (m. AMP)

The attachment sites for the m. AMP are highly conserved across all sauropsids[S12,S13]. This conservatism permits robust reconstruction of the origination and insertion sites of this muscle in all dinosaurs as level I inferences. *Plateosaurus engelhardti* exhibits a wide surface on the pterygoid wing of the quadrate for the origination of the m. AMP, as in other dinosaurs including *Diplodocus* and *Camarasaurus*[S12,S19,S20]. The muscle would then have inserted into the mandibular adductor fossa. Galton[S2] and Fairman[S16] reconstructed the m. AMP as filling the entire mandibular fossa in *Plateosaurus*; however the reconstructed insertion sites of the m.PST group herein means that the m. AMP is restricted to the caudal two-thirds of the attachment site.

m. pterygoideus dorsalis (m. PTd)

Origination and insertion sites of the m. PTd are highly conserved across sauropsids, allowing robust level I inferences of attachment sites in dinosaurs[S12,S13]. In *Plateosaurus engelhardti* the m. PTd would have originated from the lateral surface of the pterygoid flange[S2], extending dorsally onto the dorsal surface of the pterygoid, leaving a generally smooth surface. It extended at least as

far rostrally as the suture with the ectopterygoid, occupying a trough-like depression in the dorsolateral surface of the pterygoid, similar to the extent reconstructed for *Erlikosaurus*[S11].

The mandibular insertion site, along the medial border of the prearticular and articular, is also a type I inference in dinosaurs[S12]. In *Plateosaurus engelhardti* the muscle appears to have attached to the medioventral surface of the prearticular where it borders the articular fossa, extending caudally into a slight depression on the medial surface of the retroarticular process.

m. pterygoideus ventralis (m. PTv)

The attachment sites for m. PTv would have originated from a smooth edge on the ventrolateral surface of the pterygoid, extending onto the ventral edge of the pterygoid flange, although, as for the m. PTd unambiguous direct correlates of this attachment are not obvious. It then would have inserted onto the ventral edge of the angular and articular, extending into a slightly excavated area on the lateral surface of the mandible indicating that this muscle would have wrapped around the ventral edge of the jaw to insert onto the angular and surangular. Although the origination and insertion sites for this muscle are well-constrained, the muscle thickness is less so as there are no osteological or reconstructed topological constraints upon how far the muscle could have bulged medially towards the oral cavity. As a result, to make a conservative estimate, the muscle was projected to maintain a similar thickness to that reconstructed from the more well-constrained insertion site for the majority of its length.

Stegosaurus stenops

m. adductor mandibulae superficialis (m. AMES)

The m. AMES originates from the ventromedial surface of the postorbital/squamosal (supratemporal bar) in *Stegosaurus stenops*. A prominent ridge separates the ventral surface of the supratemporal bar into a medial and a lateral part. The medial part is deeply excavated and the m.

AMES most likely has a fleshy attachment here[S12]. Whether the muscle's origin extends to the lateral surface is not clear. The ridge separating the ventral surface of the supraorbital bar continues caudally onto the base of the squamosal and the quadrate contact, further separating the adductor chamber from the rostrolateral surface of the quadrate. As a result, the region near the squamosal/quadrate contact is deeply excavated and reminiscent of a muscle attachment site. An additional lateral attachment of the m. AMES is possible, although the muscle is sometimes reconstructed to be restricted to the medial surface of the supratemporal bar in dinosaurs[S12]. A similar morphology is found, though, in *Corythosaurus casuarius* and Ostrom[S21] suggested an origin of the m. AMES from the lateral surface of the squamosal, however, not without noting the unusual position. A corresponding fossa on the lateral surface of the quadrate-squamosal contact is further present in specimens of *Psittacosaurus mongoliensis* and *P. gobiensis* and an attachment has for the m. AMES has been reconstructed[S22]. Fairman[S16] suggested the presence of two muscles, m. levator anguli oris (m. LAO) and m. retractor anguli oris (m. RAO), located laterally to m. AMES and originating from the ventral portions of the postorbital and squamosal, respectively, in a muscular reconstruction of *Plateosaurus engelhardti*. These muscles are found in many lepidosaurs[S23] and have been hypothesised to be present in ankylosaurs[S24] and hadrosaurids[S21]. However, as pointed out by Holliday[S12], the muscles are not present in any extant archosaurs, making their reconstruction in dinosaurs a weak (level III') inference. Therefore it seems plausible that slips of the m. AMES also attached to the lateral fossa in *Stegosaurus stenops* as it has been reconstructed herein.

The insertion of the m. AMES on the mandible is demarcated by an elongate, shallow fossa on the lateral surface of the surangular, just rostral to the articular contact. The fossa follows the dorsal margin of the surangular and is somewhat crescent-shaped. In shape and position the insertion of the m. AMES in *Stegosaurus stenops* is similar to that of *Plateosaurus engelhardti*[S12].

m. adductor mandibulae medialis (m. AMEM)

The m. AMEM originates from the caudal surface of the supratemporal fenestra. The attachment site is a deeply excavated fossa formed by the parietal/squamosal contact. The extent of the origin is indicated by pronounced ridges dorsally, laterally and ventrally. Medially, the m. AMEM extends somewhat onto the braincase wall. A faint vertical ridge or swelling marks the border with the m. AMP.

The insertion of the m. AMEM is only weakly indicated by osteological correlates and its attachment is largely based on the position of the m. AMEP and comparisons with other dinosaurian taxa. A faint fossa on the caudomedial surface of the surangular, opposite of the insertion of the m. AMES, suggests that the m. AMEM attached at this point (Fig. 7C). Holliday[S12] identified some muscle attachments sites in the mandible of *Stegosaurus* (CM 41681), but didn't map the insertion of the m. AMEM, probably because of the same uncertainties. Unlike the other muscles located in the adductor chamber, the m. AMEM is slightly curved to pass the quadrate flange, which restricts the space in the adductor chamber caudally.

m. adductor mandibulae profundus (m. AMEP)

The origin of the m. AMEP is marked by a faint depression on the medial wall of the temporal fossa. The caudal extend of the attachment is indicated by a faint ridge, whereas the rostral extent might be demarcated by a shift in surface topology close to the parietal/postorbital suture. A distinct vertical crest indicative of the rostral extent, as found in theropod dinosaurs[S11,S12], is not present in *Stegosaurus stenops*.

The m. AMEP inserts on the dorsomedial surface of the surangular, although the exact position and extent is somewhat unclear. A shallow depression and slightly rugose area is present in on the surangular caudal to coronoid region suggesting an attachment. Holliday[S12] proposed an insertion of the m. AMEP more rostrally at the level of the external mandibular fenestra and the

coronoid eminence for *Stegosaurus* (CM 41681). However, such a rostral position would result in the muscle invading the orbital region and thus possibly to conflict with the eyeball and the ocular musculature.

m. adductor mandibulae posterior (m. AMP)

Although only weakly developed, osteological correlates indicate an origin of the m. AMP from the lateral surface of the quadrate flange. A faint horizontal swelling is present on the ventral half of the quadrate flange and likely marks the attachment of the m. AMP. A more caudally located origin or caudal extension seems unlikely as this arrangement would interfere with the course of the m. AMEP and the m. AMEM, due to the invasion of the quadrate into the adductor chamber. However, comparison with other dinosaurian taxa and information from extant phylogenetic bracketing support the origin of the m. AMP in *Stegosaurus stenops*[S12].

A prominent, circular depression on the medial surface of the surangular at the caudal portion of the mandibular adductor fossa clearly marks the insertion of the m. AMP in the lower jaw.

m. pseudotemporalis superficialis (m. PSTs)

The m. PSTs forms the rostralmost muscle of the adductor complex. It originates from the rostral wall of the temporal fossa, which shows an uneven and rough surface. The lateral and medial extent of the muscle's attachment are not clearly demarcated and inferred by the position of the adjacent adductor muscles (m. AMES and m. AMEP).

The mandibular attachment of the m. PSTs is not clearly marked by osteological correlates, but has to be inferred on the basis of the condition found in extant bracketing taxa. Consequently, the m. PSTs most likely inserts in the rostral portion of the mandibular adductor fossa in basal ornithischians[S12]. This position is supported by the spatial arrangement of the surrounding muscles

of the adductor group in *Stegosaurus stenops*, in particular the m. PSTp and the m. AMP, which are located caudal to and have to be passed by the m. PSTs.

m. pseudotemporalis profundus (m. PSTp)

The muscle attachments of the m. PSTp are generally difficult to identify as osteological correlates in extant taxa as well as in various dinosaur groups are largely unclear. While the muscle is supposed to originate from the epipterygoid in some dinosaurian clades[S12], this element has been lost in several taxa, including *Stegosaurus stenops*[S8], making the origin and the presence of the m. PSTp difficult to track. Alternative origins from the laterosphenoid or the quadrate are unlikely as no osteological correlates or prominent surface features are present. Thus the presence of the m. PSTp in *Stegosaurus stenops* is not well supported and this muscle was likely absent in most other ornithischians.

m. pterygoideus dorsalis (m. PTd)

The m. PTd originates from the dorsolateral surface of the pterygoid marked by a faint depression on the bone. Although the palatines have not been preserved with the specimen of *Stegosaurus stenops* and have only been reconstructed to a generic morphology, an additional attachment of the m. PTd on the palatines seems unlikely. The palatines are located rostral and ventral to the pterygoids and would not be available as muscle attachment.

On the mandible, the m. PTd inserts into the medial surface of the articular. The attachment is marked by a faint depression rostral to the jaw joint. Holliday[S12] identified the insertion of the m. PTd in a concordant position in *Stegosaurus* (CM 41681) and other dinosaurian taxa.

m. pterygoideus ventralis (m. PTd)

As in most dinosaursS12 the, the m. pterygoideus ventralis originates from the caudoventral edge of the pterygoid. The pterygoid shows no clear osteological correlates as in some theropods[S11,S12], but this position is supported by inference of phylogenetically bracketing taxa and the arrangement of the surrounding muscles.

The m. PTv inserts on the medioventral surface of the angular and a portion of the muscle wraps around the bone to attach onto the lateral surface of the angular. The medial insertion is marked by a smooth fossa ventral to the jaw joint and the retroarticular process, whereas a rostrocaudally elongate depression indicates the lateral insertion. The latter is dorsoventrally narrow, suggesting only a moderate expansion of the muscle on the lateral side unlike the large, bulging muscle reconstructed for other dinosaurs[S12].

ADDITIONAL DATA AND FIGURES FOR BIOMECHANICAL ANALYSES

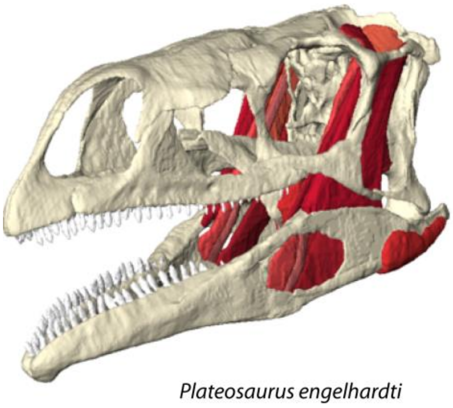

	Origin	Insertion
m. PTd	Caudodorsal surface of pterygoid	Medial surface of angular rostroventral to jaw joint
m. PTv	Ventral surface of pterygoid	Lateral and ventral surface of articular and surangular
m. PSTp	Lateral surface of laterosphenoid, basisphenoid and pterygoid region	Medial mandibular fossa
m. PSTs	Lateral surface of epipterygoid	Rostral portion of medial mandibular fossa
m. AMEP	Caudomedial surface of supratemporal fossa on parietal	Coronoid eminence
m. AMEM	Caudal surface of supratemporal fossa on squamosal/parietal bar	Rostromedial surface of coronoid
m. AMES	Medial surface of supratemporal bar on postorbital and squamosal	Dorsal surface of coronoid
m. AMP	Lateral surface of quadrate	Caudal portion of medial mandibular fossa

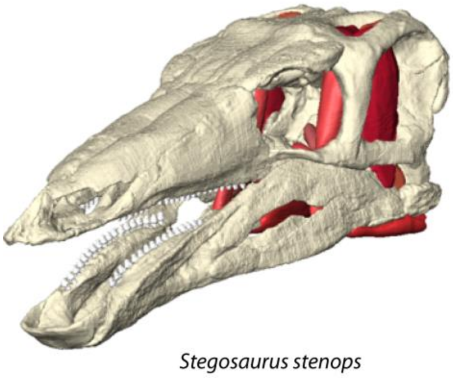

	Origin	Insertion
m. PTd	Dorsolateral surface of pterygoid	Medial surface of angular and articular rostroventral to jaw joint
m. PTv	Caudoventral surface of pterygoid	Lateral, medial and ventral surface of articular and surangular
m. PSTp	absent	absent
m. PSTs	Rostral surface of supratemporal fossa on postorbital, parietal and laterosphenoid	Rostral portion of medial mandibular fossa
m. AMEP	Medial surface of supratemporal fossa on parietal	Coronoid eminence
m. AMEM	Caudal surface of supratemporal fossa on squamosal/parietal bar	Caudomedial surface of surangular
m. AMES	Medial surface of supratemporal bar on postorbital and squamosal, squamosal fossa	Dorsolateral surface of surangular and articular
m. AMP	Lateral surface of quadrate flange	Medial mandibular fossa and medial surface of surangular

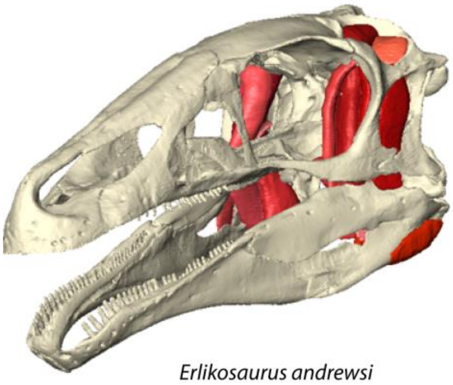

	Origin	Insertion
m. PTd	Dorsal surface of pterygoid	Medial surface of angular and articular ventral to jaw joint
m. PTv	Caudoventral surface of pterygoid	Lateral and ventral surface of articular and surangular
m. PSTp	Lateral surface of laterosphenoid, basisphenoid and pterygoid region	Medial mandibular fossa
m. PSTs	Rostral surface of supratemporal fossa on postorbital, parietal and laterosphenoid	Rostral portion of medial mandibular fossa
m. AMEP	Caudomedial surface of supratemporal fossa on parietal	Coronoid eminence
m. AMEM	Caudal surface of supratemporal fossa on squamosal/parietal bar	Dorsomedial surface of surangular
m. AMES	Medial surface of supratemporal bar on postorbital and squamosal	Dorsomlateral surface surangular
m. AMP	Lateral surface of quadrate	Medial mandibular fossa

Figure S1 Muscle attachment sites for *Plateosaurus engelhardti*, *Stegosaurus stenops* and *Erlikosaurus andrewsi*.

Muscle	Muscle force (original size)	Muscle force (scaled)	Sagittal insertion angle [°]	Coronal insertion angle [°]	contribution to bite force
			Plateosaurus engelhardti		
m. PTd	35.55	33.10	24.60	13.80	8.90
m. PTv	50.85	47.34	20.00	17.00	12.70
mPSTp	12.15	11.31	44.30	6.20	3.00
m. PSTs	13.95	12.99	53.00	1.30	3.50
m. AMEP	36.45	33.93	55.70	5.10	9.10
m. AMEM	52.20	48.60	34.60	1.50	13.10
m. AMES	130.50	121.50	24.00	3.50	32.70
m. AMP	67.50	62.84	39.00	2.00	16.90
**Sum**	**399.15**	**371.61**			
			Stegosaurus stenops		
m. PTd	175.50	163.39	47.00	21.00	11.00
m. PTv	426.15	396.75	34.00	29.00	26.70
mPSTp	69.75	64.94	14.00	44.00	4.30
m. PSTs	175.50	163.39	22.00	9.00	11.00
m. AMEP	168.75	157.11	3.00	15.00	10.60
m. AMEM	170.10	158.36	8.00	11.00	10.60
m. AMES	305.10	284.05	5.00	3.00	19.10
m. AMP	105.30	98.03	28.00	40.00	6.60
**Sum**	**1596.00**	**1485.88**			
			Erlikosaurus andrewsi		
m. PTd	55.94	-	35.50	10.30	8.80
m. PTv	182.15	-	7.20	6.40	28.90
mPSTp	13.49	-	2.00	20.90	2.90
m. PSTs	35.76	-	15.60	4.20	10.50
m. AMEP	78.11	-	17.50	16.50	18.90
m. AMEM	76.80	-	16.30	9.20	11.10
m. AMES	83.22	-	6.50	4.30	13.50
m. AMP	34.86	-	6.10	10.90	5.30

Sum	560.31				

Table S1 Calculated muscle forces and insertion angles for *Erlikosaurus andrewsi*, *Plateosaurus engelhardti* and *Stegosaurus stenops* presented in this study. Abbreviations: m. AMEM, m. adductor mandibulae externus medialis; m. AMEP, m. adductor mandibulae externus profundus; m. AMES, m. adductor mandibulae externus superficialis; m. AMP, m. adductor mandibulae posterior; m. PSTp, m. pseudotemporalis profundus; m. PSTs, m. pseudotemporalis superficialis; m. PTd, m. pterygoideus dorsalis; m. PTv, m. pterygoideus ventralis.

Taxon	FEA - Model	Number of elements
Erlikosaurus andrewsi	Skull, original size	1 905 393
	Skull with rhamphotheca, original size	1 989 380
	Lower jaw, original size	1 007 423
	Lower jaw with rhamphotheca, original size	1 077 317
Plateosaurus engelhardti	Skull, original size	2 067 955
	Lower jaw, original size	907 791
	Skull, scaled	2 015 509
	Lower jaw, scaled	1 022 293
Stegosaurus stenops	Skull, original size	2 220 304
	Lower jaw, original size	1 103 060
	Skull, scaled	2 235 813
	Lower jaw, scaled	1 022 293
	Skull with rhamphotheca, original size	2 301 925
	Lower jaw with rhamphotheca, original size	1 212 893
	Skull with antorbital fenestra, original size	2 154 543
	Skull with antorbital fenestra, scaled	1 964 692
Plant model	10 mm diameter	93 685
	5 mm diameter	35 619

Table S2 Model sizes of the individual FE models.

#	Model	Loaded forces	Constraints			Constraints simulating bite process		
		Adductor muscles	Occipital condyle	Paroccipital process	Quadrate	Tip of skull	1st maxillary tooth	Last maxillary tooth
	Erlikosaurus andrewsi	X						
1	Skull, original size	X	6	7	7	X		
2	Skull, original size	X	6	7	7		X	
3	Skull, original size	X	6	7	7			X
4	Skull and rhamphotheca, original size	X	6	7	7	X		
5	Skull and rhamphotheca, original size	X	6	7	7		X	
6	Skull and rhamphotheca, original size	X	6	7	7			X
	Plateosaurus engelhardti							
7	Skull, original size	X	6	7	7	X		
8	Skull, original size	X	6	7	7		X	
9	Skull, original size	X	6	7	7			X
10	Skull, scaled	X	6	7	7	X		
11	Skull, scaled	X	6	7	7		X	
12	Skull, scaled	X	6	7	7			X
	Stegosaurus stenops							
13	Skull, original size	X	6	7	7	X		
14	Skull, original size	X	6	7	7		X	
15	Skull, original size	X	6	7	7			X
16	Skull, scaled	X	6	7	7	X		
17	Skull, scaled	X	6	7	7		X	
18	Skull, scaled	X	6	7	7			X
19	Skull and rhamphotheca, original size	X	6	7	7	X		
20	Skull and rhamphotheca, original size	X	6	7	7		X	
21	Skull and rhamphotheca, original size	X	6	7	7			X
22	Skull with antorbital fenestra, original size	X	6	7	7	X		
23	Skull with antorbital fenestra, original size	X	6	7	7		X	
24	Skull with antorbital fenestra, original size	X	6	7	7			X
25	Skull with antorbital fenestra, scaled	X	6	7	7	X		
26	Skull with antorbital fenestra, scaled	X	6	7	7		X	

27	Skull with antorbital fenestra, scaled	X	6	7	7		X

Table S3 Model setup for different tested FE models of the skulls of *Erlikosaurus andrewsi*, *Plateosaurus engelhardti* and *Stegosaurus stenops* presented in this study.

#	Model	Loaded forces	Constraints	Constraints simulating bite process		
		Adductor muscles	Glenoid	Tip of skull	1st maxillary tooth	Last maxillary tooth
	Erlikosaurus andrewsi	X				
1	Lower jaw, original size	X	7	X		
2	Lower jaw, original size	X	7		X	
3	Lower jaw, original size	X	7			X
4	Lower jaw and rhamphotheca, original size	X	7	X		
5	Lower jaw and rhamphotheca, original size	X	7		X	
6	Lower jaw and rhamphotheca, original size	X	7			X
	Plateosaurus engelhardti					
7	Lower jaw, original size	X	7	X		
8	Lower jaw , original size	X	7		X	
9	Lower jaw , original size	X	7			X
10	Lower jaw , scaled	X	7	X		
11	Lower jaw , scaled	X	7		X	
12	Lower jaw , scaled	X	7			X
	Stegosaurus stenops					
13	Lower jaw, original size	X	7	X		
14	Lower jaw, original size	X	7		X	
15	Lower jaw, original size	X	7			X
16	Lower jaw, scaled	X	7	X		
17	Lower jaw, scaled	X	7		X	
18	Lower jaw, scaled	X	7			X
19	Lower jaw and rhamphotheca, original size	X	7	X		
20	Lower jaw and rhamphotheca, original size	X	7		X	
21	Lower jaw and rhamphotheca, original size	X	7			X

Table S4 Model setup for different tested FE models of the lower jaws of *Erlikosaurus andrewsi*, *Plateosaurus engelhardti* and *Stegosaurus stenops* presented in this study.

#	Model	Loaded forces	Taxon			Constraints simulating bite process		
		Bite force	*Plateosaurus engelhardti*	*Stegosaurus stenops*	*Erlikosaurus andrewsi*	Tip of skull	1st maxillary tooth	Last maxillary tooth
	10 mm diameter							
1	Bite at tip of skull	69 N	X			X		
2	Bite at 1st maxillary tooth	74 N	X				X	
3	Bite at last maxillary tooth	138 N	X					X
4	Bite at tip of skull	243 N		X		X		
5	Bite at 1st maxillary tooth	368 N		X			X	
6	Bite at last maxillary tooth	410 N		X				X
7	Bite at tip of skull	50 N			X	X		
8	Bite at 1st maxillary tooth	94 N			X		X	
9	Bite at last maxillary tooth	121 N			X			X
	5 mm diameter							
10	Bite at tip of skull	69 N	X			X		
11	Bite at 1st maxillary tooth	74 N	X				X	
12	Bite at last maxillary tooth	138 N	X					X
13	Bite at tip of skull	243 N		X		X		
14	Bite at 1st maxillary tooth	368 N		X			X	
15	Bite at last maxillary tooth	410 N		X				X
16	Bite at tip of skull	50 N			X	X		
17	Bite at 1st maxillary tooth	94 N			X		X	
18	Bite at last maxillary tooth	121 N			X			X
	10 mm diameter (scaled bite forces)							
19	Bite at tip of skull	46 N	X			X		
20	Bite at 1st maxillary tooth	49 N	X				X	
21	Bite at last maxillary tooth	123 N	X					X
22	Bite at tip of skull	167 N		X		X		
23	Bite at 1st maxillary tooth	201 N		X			X	
24	Bite at last maxillary tooth	321 N		X				X
	5 mm diameter (scaled bite forces)							
25	Bite at tip of skull	46 N	X			X		
26	Bite at 1st maxillary tooth	49 N	X				X	

27	Bite at last maxillary tooth	123 N	X				X
28	Bite at tip of skull	167 N		X	X		
29	Bite at 1st maxillary tooth	201 N		X		X	
30	Bite at last maxillary tooth	321 N		X			X

Table S5 Model setup for different tested FE plant models.

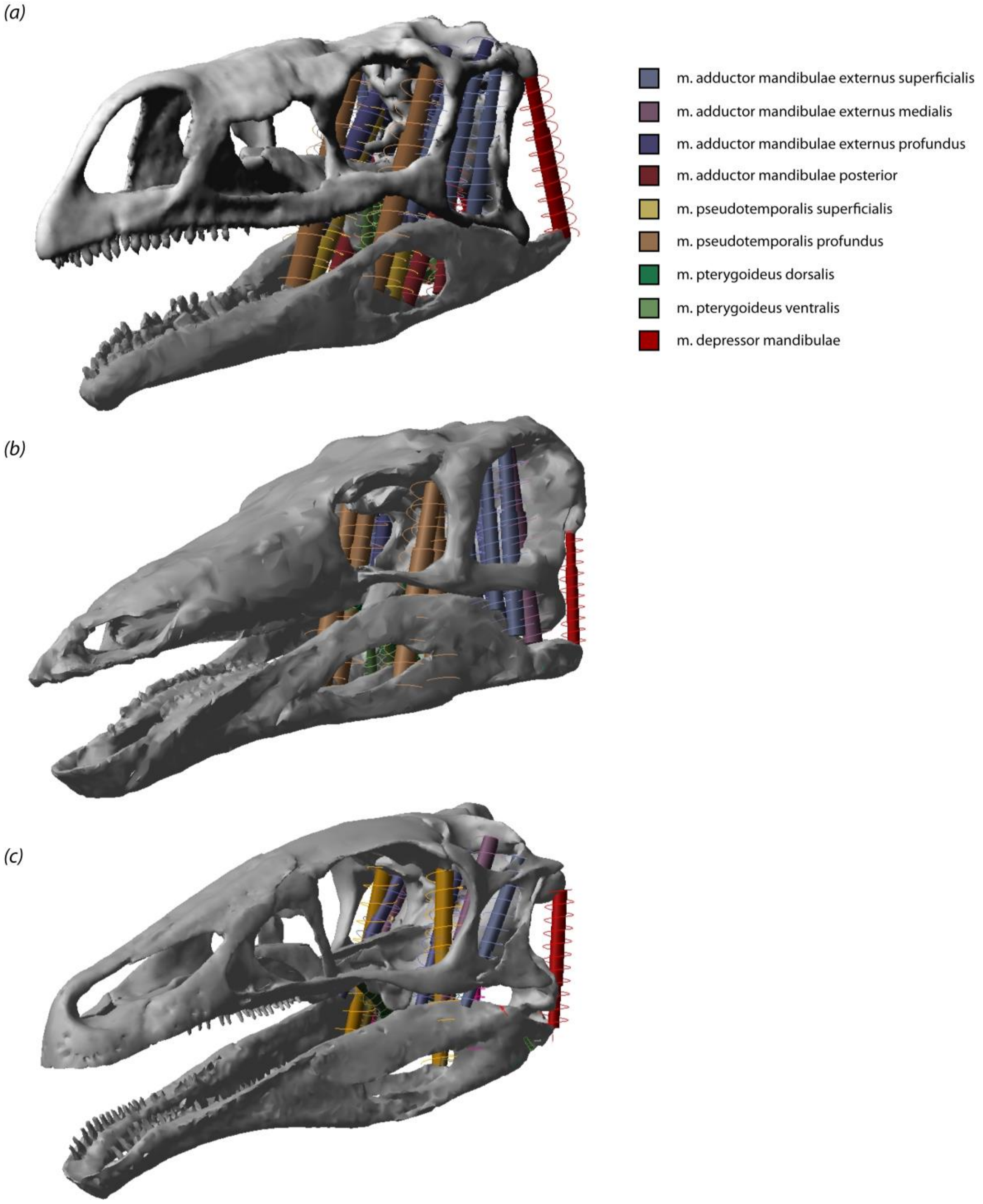

Figure S2 Multibody dynamics model of *(a) Plateosaurus engelhardti*, *(b) Stegosaurus stenops* and *(c) Erlikosaurus andrewsi*.

	Skull	Lower jaw
1	Tip of skull/premaxillae contact	Symphyseal contact dorsal
2	Premaxilla-maxilla contact ventral right	Symphyseal contact ventral
3	Premaxilla-maxilla contact ventral left	1st tooth right
4	Rostral corner of naris right	1st tooth left
5	Rostral corner of naris left	5th tooth right
6	Nasal-frontal contact midpoint	5th tooth left
7	Nasal-frontal contact right	10th tooth right
8	Nasal-frontal contact left	10th tooth left
19	First maxillary tooth right	last tooth right
10	First maxillary tooth left	last tooth left
11	8th maxillary tooth right	Surangular contact right
12	8th maxillary tooth left	Surangular contact left
13	Last maxillary tooth right	Prearticular contact right
14	Last maxillary tooth left	Prearticular contact left
15	Jugal-lacrimal-maxilla contact right	Rostral corner fenestra right
16	Jugal-lacrimal-maxilla contact left	Rostral corner fenestra left
17	Frontal-lacrimal contact right	Caudal corner fenestra right
18	Frontal-lacrimal contact left	Caudal corner fenestra left
19	Parietal-frontal contact midpoint	Surangular-articular contact right
20	Frontal-postorbital contact right	Surangular-articular contact left
21	Frontal-postorbital contact left	Retroarticular process right
22	Rostroventral corner of temporal fenestra right	Retroarticular process left
23	Rostroventral corner of temporal fenestra left	Medial expansion articular right
24	Quadrate condyle lateral right	Medial expansion articular left
25	Quadrate condyle lateral left	
26	Quadrate condyle medial right	
27	Quadrate condyle medial left	

28	Paroccipital process tip right	
29	Paroccipital process tip left	
30	Condyle midpoint dorsal	
31	Ectopterygoid process right	
32	Ectopterygoid process left	
33	Vomer caudal end	
34	Vomer rostral end	

Table S6 Landmark definitions for skull and lower jaw models of *Erlikosaurus andrewsi*, *Plateosaurus engelhardti* and *Stegosaurus stenops* presented in this study.

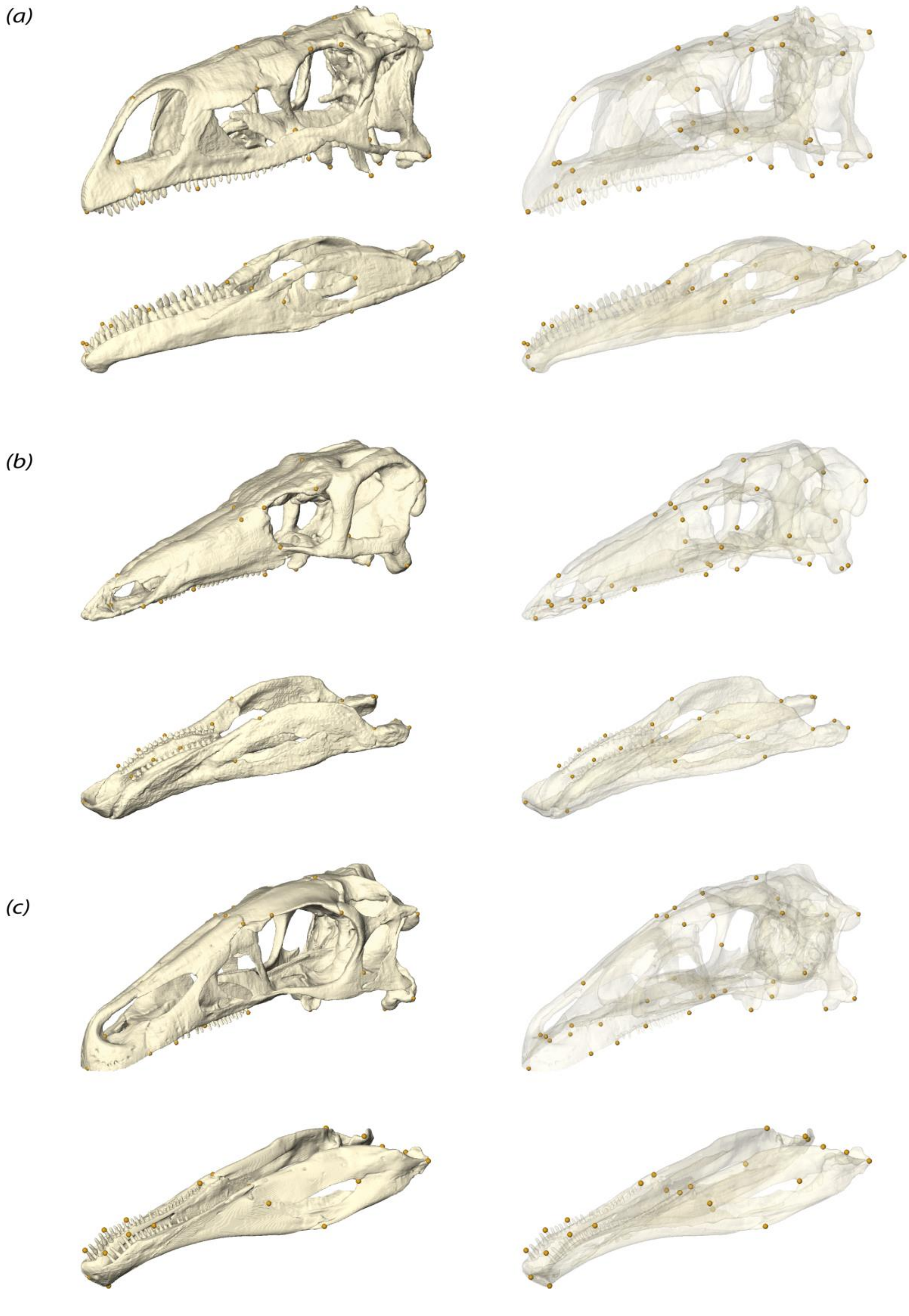

Figure S3 Assigned landmarks for skull and lower jaw models. *(a) Plateosaurus engelhardti*, *(b) Stegosaurus stenops* and *(c) Erlikosaurus andrewsi*.

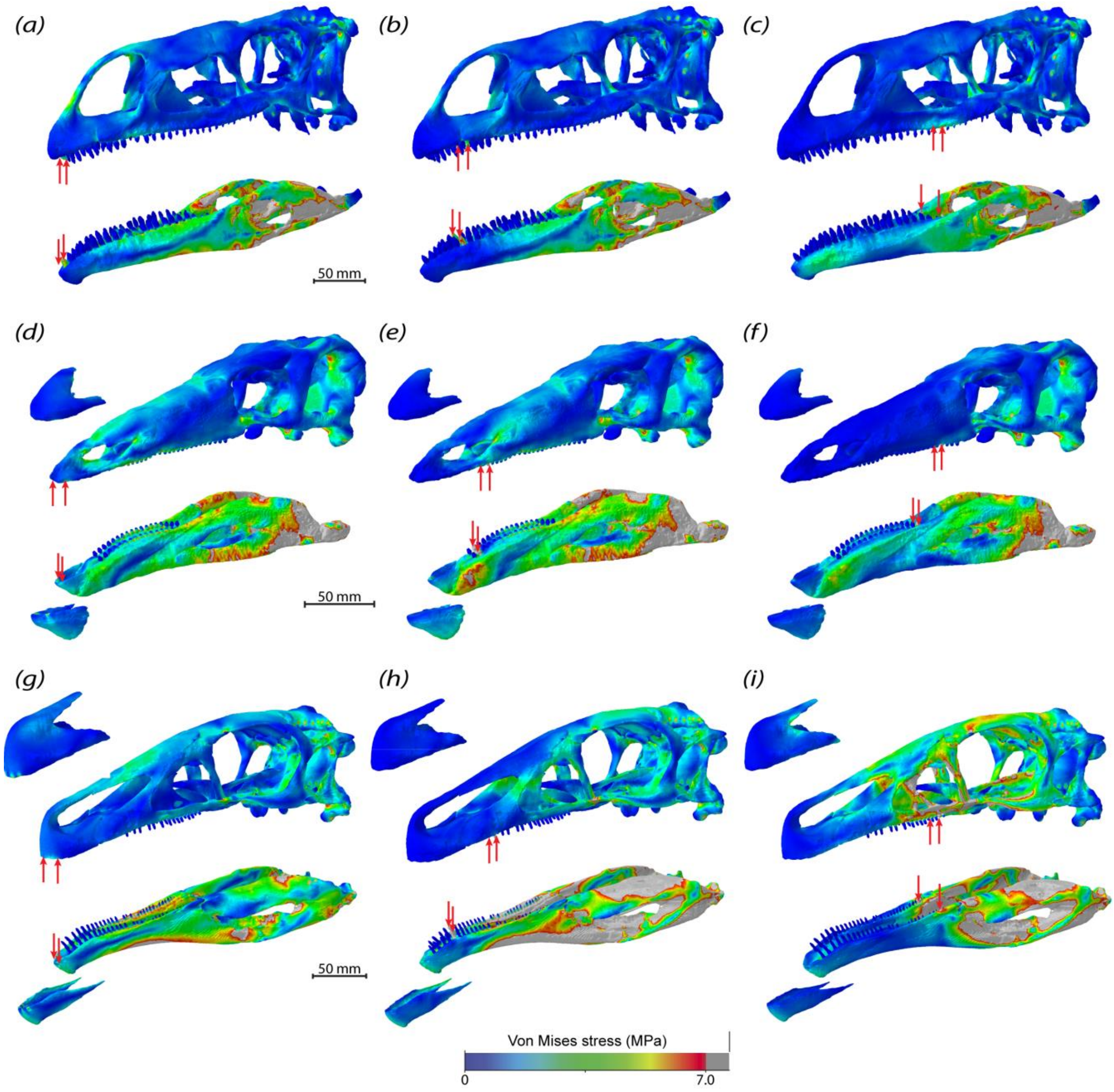

Figure S4 Comparison of Von Mises stress distribution for models incorporating keratinous structures. Models of *(a-c) Plateosaurus engelhardti*, *(d-f) Stegosaurus stenops* and *(g-i) Erlikosaurus andrewsi* subjected to different bite scenarios. From left to right, bilateral bite at the tip of the skull/dentary, the first maxillary tooth/occluding tooth on dentary, last occluding maxillary/dentary tooth (indicated by red arrows). All models in original size, but scaled to same peak stress.

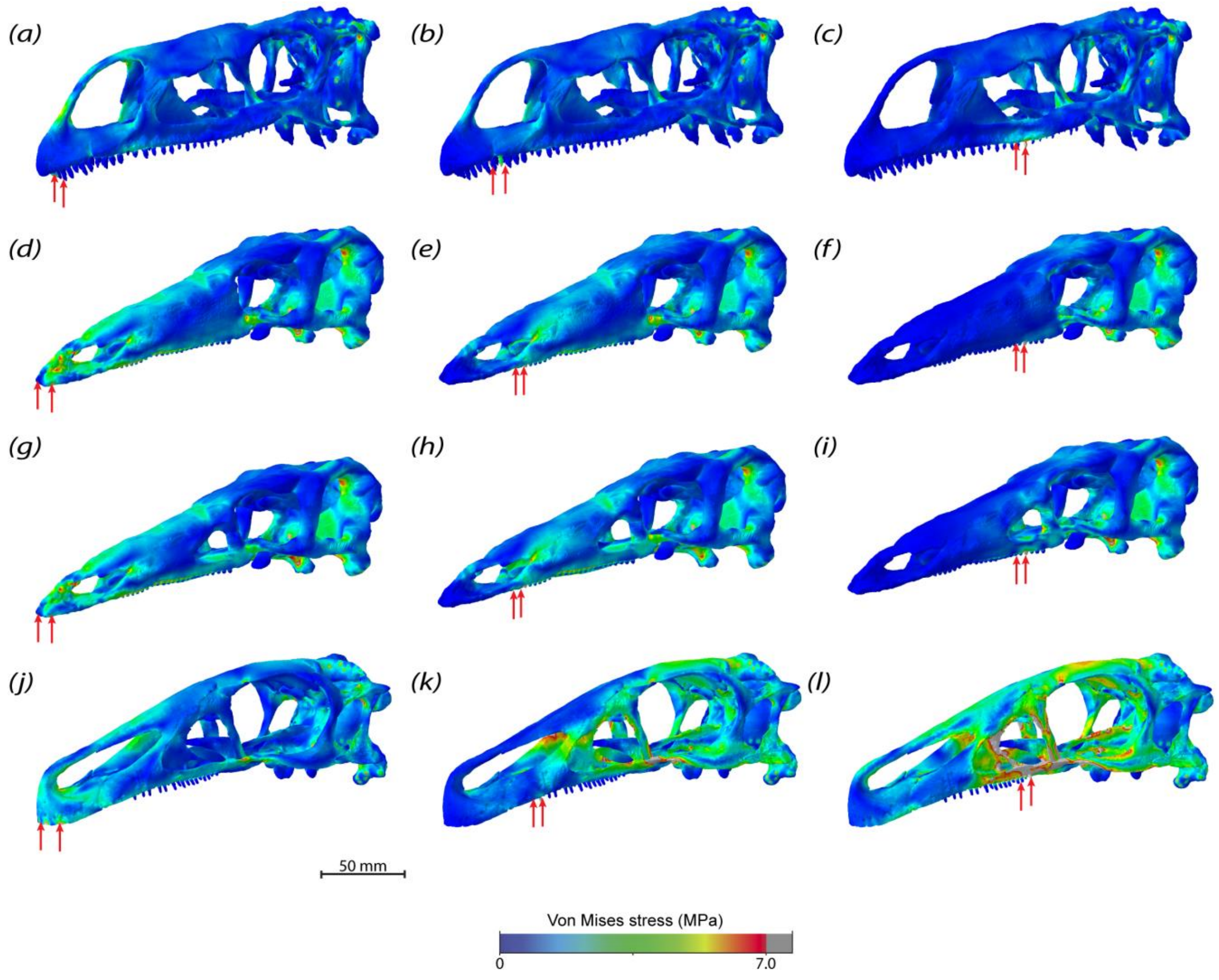

Figure S5 Comparison of Von Mises stress distribution for models an antorbital fenestra. Models of *(a-c) Plateosaurus engelhardti*, *(d-f) Stegosaurus stenops* without and *(g-i)* with antorbital fenestra, *(j-l) Erlikosaurus andrewsi* subjected to different bite scenarios. From left to right, bilateral bite at the tip of the skull/dentary, the first maxillary tooth/occluding tooth on dentary, last occluding maxillary/dentary tooth (indicated by red arrows). All models scaled to same surface area and to same peak stress.

SUPPLEMENTAL REFERENCES

S1. Galton, P.M. (1984). Cranial anatomy of the prosauropod dinosaur *Plateosaurus* from the Knollenmergel (Middle Keuper, Upper Triassic) of Germany. II. Geologica et Palaeontologica *18*, 139-171.

S2. Galton, P.M. (1985). Cranial anatomy of the prosauropod dinosaur *Plateosaurus* from the Knollenmergel (Middle Keuper, Upper Triassic) of Germany. II. Geologica et Palaeontologica, *19*,119-159.

S3. Wilson, J.A. and Sereno, P.C. (1998). Early evolution and higher-level phylogeny of sauropod dinosaurs. Society of Vertebrate Paleontology Memoirs, *5*, 1-68.

S4. Yates, A.M. (2003). The species of taxonomy of the sauropodomorph dinosaurs from the Löwenstein Formation (Norian, Late Triassic) of Germany. Palaeontology, *46*, 317-337.

S5. Galton, P.M., and Upchurch, P. (2004). Prosauropoda. In The Dinosauria, D.B. Weishampel, P. Dodson and H. Osmolska, eds. (Berkeley and Los Angeles: University of California Press), pp. 232-258.

S6. Prieto-Marquez, A., and Norell, M. A. (2011). Redescription of a Nearly Complete Skull of *Plateosaurus* (Dinosauria: Sauropodomorpha) from the Late Triassic of Trossingen (Germany). American Museum Novitates, *3727*, 1-58.

S7. Wiley, D.F., Amenta, N., Alcantara, D.A., Ghosh, D., Kil, Y.J., Delson, E., Harcourt-Smith, W., Rohlf, F.J., St John, K., and Hamann, B. (2005). Evolutionary morphing. Visualization, 2005. VIS 05. IEEE, 431-438.

S8. Gilmore, C.W. (1914). Osteology of the armored dinosaurs in the United States National Museum, with special reference to the genus *Stegosaurus*. Bulletin of the US National Museum, *89*, 1-136.

S9. Galton, P.M., and Upchurch, P. (2004). Stegosauria. In The Dinosauria, 2nd Edition, D.B. Weishampel, P. Dodson and H. Osmolska, eds. (Berkeley, CA: University of California Press), pp. 343-362.

S10. Lautenschlager, S., Witmer, L.M., Altangerel, P., Zanno, L.E., and Rayfield, E.J. (2014). Cranial anatomy of *Erlikosaurus andrewsi* (Dinosauria, Therizinosauria): new insights based on digital reconstruction. Journal of Vertebrate Paleontology *34*, 1263-1291.

S11. Lautenschlager, S. (2013). Cranial myology and bite force performance of *Erlikosaurus andrewsi*: a novel approach for digital muscle reconstructions. Journal of Anatomy, *222*, 260-272.

S12. Holliday, C.M. (2009). New insights into dinosaur jaw muscle anatomy. The Anatomical Record, *292*, 1246-1265.

S13. Holliday, C.M. And Witmer, L.M. (2007). Archosaur adductor chamber evolution: integration of musculoskeletal and topological criteria in jaw muscle homology. Journal of Morphology, *268*, 457-484.

S14. Coria, R.A., Currie, P.J. (2003). The braincase of *Giganotosaurus carolinii* (Dinosauria: Theropoda) from the upper Cretaceous of Argentina. Journal of Vertebrate Paleontology, *22*, 802-811.

S15. Molnar, R.E (2008). Reconstruction of the jaw musculature of *Tyrannosaurus rex*. *Tyrannosaurus Rex*, the Tyrant King, 1-255.

S16. Fairman, J.E. (1999). Prosauropod and iguanid jaw musculature: a study of the evolution of form and function [Unpublished Masters Thesis]. Baltimore: Johns Hopkins University. 1-94 p.

S17. Tsai, H.P. and Holliday, C.M. (2011). Ontogeny of the *Alligator* cartilago transiliens and its significance for sauropsid jaw muscle evolution. PLoS ONE, *6*, e24935

S18. Holliday, C.M., Tsai, H.P., Skiljan, R.J., George, I.D. And Pathan, S. (2013). A 3D Interactive Model and Atlas of the Jaw Musculature of *Alligator mississippiensis*. PLoS ONE, *8*, e62806.

S19. Young, M.T., Rayfield, E.J., Holliday, C.M., Witmer, L.M., Button, D.J., Upchurch, P. and Barrett, P.M. (2012). Cranial biomechanics of *Diplodocus* (Dinosauria, Sauropoda): testing hypotheses of feeding behaviour in an extinct megaherbivore. Naturwissenschaften, *99*, 637-643.

S20. Button, D.J., E.J. Rayfield, and P.M. Barrett (2014). Cranial biomechanics underpins high sauropod diversity in resource-poor environments. Proceedings of the Royal Society B, Biological Sciences, *281*, 20142114.

S21. Ostrom, J.H. (1961). Cranial morphology of the hadrosaurian dinosaurs of North America. Bulletin of the American Museum of Natural History, *122*, 33-186.

S22. Sereno, P.C., Xijin, Z., Lin, T. (2010). A new psittacosaur from Inner Mongolia and the parrot-like structure and function of the psittacosaur skull. Proceedings of the Royal Society B, Biological Sciences, *277*, 199-209.

S23. Daza, J.D., Diogo, R., Johnston, P., Abdala, V. (2011). Jaw adductor muscles across lepidosaurs: a reappraisal. The Anatomical Record, *294*, 1765-1782.

S24. Haas, G. (1969). On the jaw muscles of ankylosaurs. American Museum Novitates, *2399*, 1-12.
